# Utilisation of outpatient medical services by people with diagnosed diabetes during the COVID-19 pandemic in Germany

**DOI:** 10.25646/8333

**Published:** 2021-06-16

**Authors:** Yong Du, Jens Baumert, Stefan Damerow, Alexander Rommel, Christa Scheidt-Nave, Christin Heidemann

**Affiliations:** Robert Koch Institute, Berlin, Department of Epidemiology and Health Monitoring

**Keywords:** UTILISATION, MEDICAL SERVICES, DIABETES, SARS-COV-2, COVID-19, GEDA

## Abstract

People with diabetes regularly need outpatient medical care due to their disease and possible concomitant and secondary illnesses. Using data from the nationwide GEDA 2019/2020-EHIS survey conducted from April 2019 to September 2020, the present study examines developments in outpatient utilisation behaviour during the measures put in place to contain the SARS-CoV-2 pandemic. During the observation period, people with diabetes had a significantly higher rate of utilisation of medical services provided by general practitioners (GPs) and specialists than the population as a whole. In the spring of 2020, when the restrictions were put in place, utilisation of specialist medical services by people with diabetes decreased temporarily by 46% compared to the 2019 reference period. In contrast, no relevant decline in the utilisation of medical services provided by GPs was observed, but this could be related to adaptations of care provision through telephone consultations for people with regularly requiring GP office visits. The issue examined here requires further observations in view of the renewed containment measures.

## Introduction

Since the beginning of 2020, SARS-CoV-2 has also been spreading in Germany. In addition to general hygiene measures, observing social distancing rules and wearing a mouth and nose cover, certain phases of stricter contact restrictions have been put in place during the pandemic as part of the non-pharmaceutical measures aiming at containing SARS-CoV-2 infection. In March 2020, the German government in agreement with the federal states decided to implement comprehensive measures for infection control and capacity redistribution for outpatient and inpatient care in addition to general contact restrictions. These measures were gradually phased out between the end of April 2020 and the end of October 2020, but were put in place again in November 2020 [1]. Accounting data from the statutory health insurance (SHI) and the Associations of SHI Physicians (Kassen ärztliche Vereinigungen) show that these measures have had an impact on the provision of outpatient and inpatient care [2–4]. However, until now, very limited information has been available on utilisation behaviour by people with chronic illnesses [4, 5]. Results from the closely conducted telephone-based COSMO (COVID-19 Snapshot Monitoring) survey indicate that the majority of adults with chronic illnesses were able to attend necessary doctor’s appointments and receive necessary medications largely without restrictions during the first stage of the containment measures [5].

People with diabetes mellitus belong to the group of chronically ill people and need continuous medical care. They also belong to the risk group for severe courses of COVID-19 [6–9]. This particularly applies to those who have poor control of their blood sugar levels or complications, regardless of whether they have type 1 or type 2 diabetes [6]. Fear of being infected with SARS-CoV-2 and changes in the health care delivery may have led people with diabetes to avoid visiting a doctor’s office. Further, it is important to understand how long such changes in health care utilisation have lasted.

The present study uses data from the nationwide, population-based GEDA 2019/2020-EHIS study to help answer the following questions: (1) How has the use of general practitioner (GP) and specialist services developed among people with diabetes during the observation period from April 2019 to September 2020? and (2) Was there a reduction in health care utilisation among people with diabetes during the containment measure period in the spring of 2020 compared to the corresponding period in 2019?

**GEDA 2019/2020-EHIS**

Fifth follow-up survey of the German Health Update

**Data holder:** Robert Koch Institute

**Objectives:** Provision of reliable information on the health status, health behaviour and health care of the population living in Germany, with the possibility of European comparisons

**Study design**: Cross-sectional telephone survey

**Population:** German-speaking population aged 15 and older living in private households that can be reached via landline or mobile phone

**Sampling:** Random sample of landline and mobile telephone numbers (dual-frame method) from the ADM sampling system (Arbeitskreis Deutscher Markt- und Sozialforschungsinstitute e.V.)

**Sample size:** 23,001 respondents

**Study period:** April 2019 to September 2020

**GEDA survey waves:**

▶ GEDA 2009
▶ GEDA 2010
▶ GEDA 2012
▶ GEDA 2014/2015-EHIS
▶ GEDA 2019/2020-EHIS

Further information in German is available at www.geda-studie.de

## Indicator

Definition of the indicators on the utilisation of outpatient GP and specialist services was based on the answers of participants to the questions about visits to GPs and specialists in the four weeks prior to the interview. Information on the utilisation of medical services provided by GPs was collected using the question ‘How often have you consulted a GP in the last 4 weeks for advice, an examination or treatment?’; this question was accompanied by the following note: ‘Please include visits to medical practices, home visits and consultations provided over the telephone’. Data on the utilisation of specialist medical services was collected using an adapted version of the same question and was accompanied by the following note: ‘This does not include visits to a dentist or general practitioner’. The answers provided were used to establish two dichotomous variables which differentiated between people who had or had not used medical services provided by a GP or specialist. Diabetes status was determined using answers from the question ‘Have you had any of the following illnesses or complaints in the last 12 months?’. The answer categories provided a list of diseases and conditions, one of which was ‘diabetes (not including gestational diabetes)’. The survey did not collect any data about diabetes type, duration of illness, treatment (insulin, tablets and combinations) or diabetes-specific complications.

The following analyses are based on data from GEDA 2019/2020-EHIS, which was carried out as a telephone survey of the resident population in Germany aged 15 years or above [10]. The sample comprised 23,001 individuals who participated in the survey during the period of April 2019 to September 2020. No information was available for 192 participants on diabetes status or utilisation of medical services provided by GPs or specialists. Of the remaining 22,809 participants, a total of 2,044 (938 women, 1,106 men) who reported diabetes in the last twelve months were included in this analysis. A period of the twelve calendar weeks (CW) from CW 15 to CW 26 (i.e. beginning of April through end of June) in 2020 was considered as the time period of containment measures. Since utilisation of medical services can be affected by the seasons, the same period from CW 15 to CW 26 in 2019 was used as comparison period to examine the potential impact of the pandemic on the utilisation of medical services [10]. The analysis for health care utilisation from CW 15 to CW 26 are based on the responses of 256 people with diabetes in 2019 and 351 people with diabetes in 2020.

In order to map possible changes in the utilisation of outpatient GP and specialist medical services during the observation period from April 2019 to September 2020, three logistic regression models were fitted each with age, sex, education and federal state as independent control variables (adjustment). A detailed description of the modelling undertaken and the software used has been published elsewhere [10]. The models allow estimation of adjusted proportions and 95% confidence intervals (CI) for the utilisation of GP and specialists medical services monthly (model 1) and weekly (smoothed, i.e. weekly fluctuations adjusted in proportions, model 2) for the entire observation period from April (CW 14) 2019 to September (CW 36) 2020 as well as for the comparison periods from CW 15 to 26 in 2019 and 2020 (model 3). A significant difference between the comparison periods is assumed if the p-value of the binary variable used to differentiate between the periods is <0.05. The analysis was done using survey procedures. A weighting factor was used throughout the analysis, which corrected deviations from the population structure in terms of the distribution of age, sex, federal state and district type as of 31 December 2018 as well as the distribution of education according to the International Standard Classification of Education (ISCED classification) in the 2018 microcensus; the survey dates before and after the containment measures came into force were also taken into account here [10].

## Results and discussion

During the observation period of April 2019 to September 2020, a total of 58.1% (95% CI 54.5%–61.7%) of respondents who had diabetes in the last twelve months used medical services provided by a GP in the last four weeks prior to their interview, 32.9% (95% CI 29.8%–36.1%) used medical services provided by a specialist.

**Figure 1:**
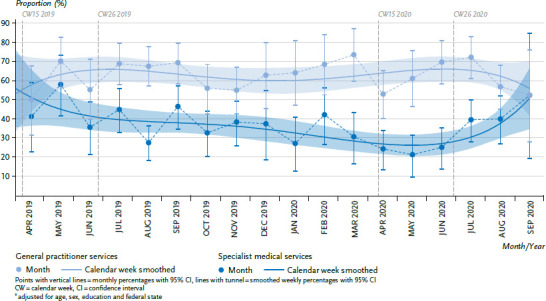
Outpatient medical services utilisation in the last four weeks by people with diabetes in the observation period between April 2019 and September 2020 (adjusted proportions^*^) Source: GEDA 2019/2020-EHIS

Figure 1 demonstrates fluctuations in the monthly figures on the utilisation of medical services provided by GPs and specialists for people with diabetes. In addition to the seasonal fluctuations that are also seen among the general population [10], the monthly fluctuations are at least partly due to the relatively low number of cases available for this study. In terms of the utilisation of medical services provided by GPs, one of the highest monthly values can be seen in March 2020; this is followed by a clear, short-term decline in April 2020 to one of the lowest monthly values, before an equally clear, rapid increase in utilisation from May 2020. This results in an overall relatively horizontal smoothed curve for the period from CW 15 to 26 of 2020. In contrast, a very low monthly value in April 2020 is followed by similarly low monthly values in May and June 2020 for the utilisation of specialist medical services; then it increases again from July 2020. This is reflected in the lowest point of the smoothed curve for the period from CW 15 to 26 of 2020. A comparison of CW 15 to 26 in 2019 and 2020 found no statistically significant difference in the adjusted proportions of utilisation of medical services provided by GPs (58.8%, 95% CI 50.3%–67.3% vs. 62.0%, 95% CI 54.8%–69.3%). However, it did highlight a significant reduction in the utilisation of specialist services, which dropped by 46% from 43.4% (95% CI 34.4%–52, 3%) to 23.6% (95% CI 17.6%–29.7%).

In a previous, comparable study conducted among the general population, the proportion for the utilisation of medical services provided by GPs or specialists in the period from April 2019 to September 2020 was consistently lower [10] than that among people with diabetes in the present study. In addition, a comparison of CW 15 to 26 in 2019 and 2020 based on the general population observed a temporary reduction in the utilisation of medical services provided by GPs (38.4% vs. 29.7%) and specialists (30.0% vs. 17.7%) [10]. The overall significantly higher rate of utilisation by people with diabetes than by the population as a whole is plausible due to their continuous need for health care. Further, people with diabetes also tend to be older, and thus have more health problems. In the general population, the smoothed curves for GP and specialist service utilisation run almost parallel to one another throughout almost the entire observation period with each curve reaching its lowest point within the period of containment measures. Among people with diabetes, with the exception of the ends of the smoothed curves (as these values are difficult to interpret due to small number of cases and resulting broad confidence intervals), few changes were observed in the utilisation of medical services provided by GPs, whereas marked changes are identifiable in the case of specialist medical services utilisation. One possible explanation for this is that necessary visits to GP office for the immediate care of diabetes were also largely made during the pandemic [11] or could have been replaced by telephone consultations (and this was also specifically mentioned in the question used for data collection). This is consistent with the observations in the COSMO study that most people with chronic diseases were still able to make the necessary visits to doctors and received the necessary medication [5]. Additional specialist visits are more likely to be occasion-based, such as if complications occur or to carry out guideline-based preventive measures (e.g. examinations of the ocular fundus every one or two years as part of the diabetes disease management programmes that have been operating in Germany since 2003/2004 [12]). Even if the results of this analysis need to be interpreted with caution due to the moderate number of cases available for each month, accounting data from the Associations of SHI Physicians (Kassenärztliche Vereinigungen) also demonstrate that the sharpest decreases occurred at the beginning of the pandemic compared to the corresponding period of the previous year, especially in terms of specialist medical care that required direct contact with patients [3]. Alongside the decrease in treatment involving direct contact until the end of May 2020, these data also indicate a rise in telephone and video consultations, which was interpreted by the authors as an adaption in the provision of care [3]. However, specialist medical examinations (e.g. of the ocular fundus and neurological examinations) cannot be carried out in this manner [13]. From the end of May 2020, therefore, accounting data also indicates that direct face-to-face contacts to patients increased again across all medical specialty groups.

In summary, during the first phase of the measures put in place to contain the SARS-CoV-2 pandemic (spring 2020), utilisation of medical services provided by GPs and specialists to the general population temporarily decreased compared to the same period in 2019. In contrast, utilisation of medical services provided by GPs to people with diabetes remained at a similar level to 2019, and this may be due to fewer patients forgoing their regular treatment, or the use of telephone consultations. In the spring of 2020, utilisation of medical services provided by specialists to people with diabetes decreased by 46% compared to the corresponding period in 2019; however, it rose again quickly from July 2020, which may indicate that patients began to visit practices again. Further observation of utilisation-related behaviour, but also of the self-assessed quality of care of diabetes and other chronic diseases, will be required during the further course of the pandemic. This is important in order to identify recurring interruption in health care utilisation, health-related and subjectively-perceived impairments, and to provide appropriate needs-based care for people with chronic illnesses.

## Key statements

- The utilisation of specialist medical services by people with diabetes significantly declined during the containment period.
- The utilisation of specialist medical services by people with diabetes began to increase again once the measures were relaxed.
- No significant decline in the utilisation of medical services provided by GPs among people with diabetes was observed during the containment period.
- People with diabetes have a much higher proportion of utilising medical services provided by GPs and specialists than the general population – this also applied during the containment period.
